# Circulating H3Cit is elevated in a human model of endotoxemia and can be detected bound to microvesicles

**DOI:** 10.1038/s41598-018-31013-4

**Published:** 2018-08-23

**Authors:** Sofie Paues Göranson, Charlotte Thålin, Annika Lundström, Lars Hållström, Julie Lasselin, Håkan Wallén, Anne Soop, Fariborz Mobarrez

**Affiliations:** 1 Karolinska Institutet, Department of Clinical Sciences, Danderyd Hospital, Division of Anaesthesia and Intensive care, 182 88 Stockholm, Sweden; 2 Karolinska Institutet, Department of Clinical Sciences, Danderyd Hospital, Division of Internal Medicine, 182 88 Stockholm, Sweden; 3 Department of Clinical Science Intervention and Technology, Karolinska University Hospital, Huddinge, Sweden; 4 Stress Research Institute, Stockholm University, Frescati Hagväg 16A, 106 91 Stockholm, Sweden; 5 Department of Clinical Neuroscience, Division for Psychology, Karolinska Institutet, Nobels väg 9, 171 65 Solna, Stockholm Sweden; 6 Karolinska Institutet, Department of Clinical Sciences, Danderyds Hospital, Division of Cardiovascular Medicine, 182 88 Stockholm, Sweden; 7 Unit of Rheumatology, Department of Medicine, Solna, Karolinska Institutet, Karolinska University Hospital, SE-171 76 Stockholm, Sweden; 8 Sophiahemmet University, Praktikertjänst Anestesi, Box 5286 102 46 Stockholm, Sweden

## Abstract

Early diagnosis of sepsis is crucial since prompt interventions decrease mortality. Citrullinated histone H3 (H3Cit), released from neutrophil extracellular traps (NETs) upon binding of platelets to neutrophils following endotoxin stimulation, has recently been proposed a promising blood biomarker in sepsis. Moreover, microvesicles (MVs), which are released during cell activation and apoptosis and carry a variety of proteins from their parental cells, have also been shown to be elevated in sepsis. In a randomized and placebo-controlled human model of endotoxemia (lipopolysaccharide injection; LPS), we now report significant LPS-induced elevations of circulating H3Cit in 22 healthy individuals. We detected elevations of circulating H3Cit by enzyme-linked immunosorbent assay (ELISA), as well as bound to MVs quantified by flow cytometry. H3Cit-bearing MVs expressed neutrophil and/or platelet surface markers, indicating platelet-neutrophil interactions. In addition, *in vitro* experiments revealed that H3Cit can bind to phosphatidylserine exposed on platelet derived MVs. Taken together; our results demonstrate that NETs can be detected in peripheral blood during endotoxemia by two distinct H3Cit-specific methods. Furthermore, we propose a previously unrecognized mechanism by which H3Cit may be disseminated throughout the vasculature by the binding to MVs.

## Introduction

Despite advances in intensive care, sepsis remains life threatening, with a 20–30% mortality rate^1,2^. Early identification of sepsis is challenging but crucial, since prompt interventions have been shown to improve survival^3–5^. Sepsis is defined as a life-threatening organ dysfunction induced by an exacerbated immune response to infection^6^, with an intense cellular activation, including neutrophil activation^7^. This severe inflammatory response can, however, also be seen in non-infectious conditions, urging the need for diagnostic tools to distinguish sepsis in order to allow for the prompt and correct use of antibiotics. As such, biomarkers used for prediction and early diagnosis, as well as for prognosis, are needed.

The neutrophil release of decondensed and web-like nuclear chromatin, termed neutrophil extracellular traps (NETs), was first described over a decade ago^8^ as part of the innate immune response against invading pathogens. Driven by lipopolysaccharide (LPS), an endotoxin found in the outer membrane of Gram-negative bacteria, NETs were observed to entrap and kill microorganisms. The mechanisms triggering LPS-induced NETosis are partly unknown, but platelets have been ascribed a central role through their binding to neutrophils following LPS-stimulation of toll-like receptors (TLR)^9^. Although NETs are considered protective in the initial stages of infection, they have been associated with detrimental effects on the host, such as the promotion of sepsis-induced coagulopathy^10–14^ and tissue and organ damage^15–17^. Emerging research has now demonstrated markers associated with NETs, such as cell free DNA (cfDNA), nucleosomes, and the antimicrobial peptides attached to the NETs upon extrusion in the blood stream of both experimental^15,16,18–21^ and clinical sepsis^9,11–13,15,22–25^. These markers are, however, not NET specific, as they can be elevated in the circulation upon conditions not related to NETosis, such as necrosis, apoptosis^26,27^ and neutrophil activation without NET formation^28,29^. Citrullinated histone H3 (H3Cit) has in this context achieved emerging interest, considered a more specific NET-marker due to the crucial role of histone citrullination in NETosis^30–32^. Upon strong neutrophil activation, the enzyme peptidylarginine deaminase (PAD4) enters the nucleus and citrullinates histone H3, leading to chromatin decondensation; the initial step of NETosis. An H3Cit specific antibody has therefore been used in microscopic immune-detection of NETs and in assessing *in vitro* neutrophil generation of NETs, although the quantification of *in vivo* circulating H3Cit has been challenging. H3Cit has, however, been detected both in murine plasma by ELISA^33^ and western blot^18,19^, as well as in the blood of critically ill and septic patients by western blot^34^ and immunofluorescence^35^. We recently detected H3Cit by a novel ELISA in a small number of plasma samples in a human model of LPS-induced endotoxemia^36^. The same ELISA furthermore recently detected plasma H3Cit in cancer patients^37,38^.

Other biomarkers shown to be elevated in sepsis are microvesicles (MVs)^39,40^, which are released from the cell membrane^41^ during cell activation and apoptosis. These vesicles are between 0.1–1.0 µm in diameter and express a variety of biologically active molecules with pro-inflammatory and pro-coagulant effects^42^. It has previously been shown that LPS administration in healthy volunteers increase plasma levels of MVs derived from platelets, leukocytes and endothelial cells^43^. In the same study, expression of the nuclear protein high-mobility group box 1 (HMGB1) was detected on platelet and monocyte derived MVs, indicating that MVs could be a source of extracellular HMGB1 and other nuclear molecules in the blood during inflammation^43^. Upon formation, MVs can also expose the negatively charged phospholipid phosphatidylserine (PS)^41,42^ and could thereby possibly bind electrostatically to the positively charged H3Cit. The role of MVs in the transportation and dissemination of NET components, such as H3Cit, has, however, not yet been studied.

The aim of this study was to determine the effect of LPS on circulating H3Cit in a human model of endotoxemia and to investigate a possible presence and cellular origin of H3Cit-bearing MVs.

## Results

### Circulating H3Cit levels are elevated after LPS injection in a human model of endotoxemia

**Figure 1:**
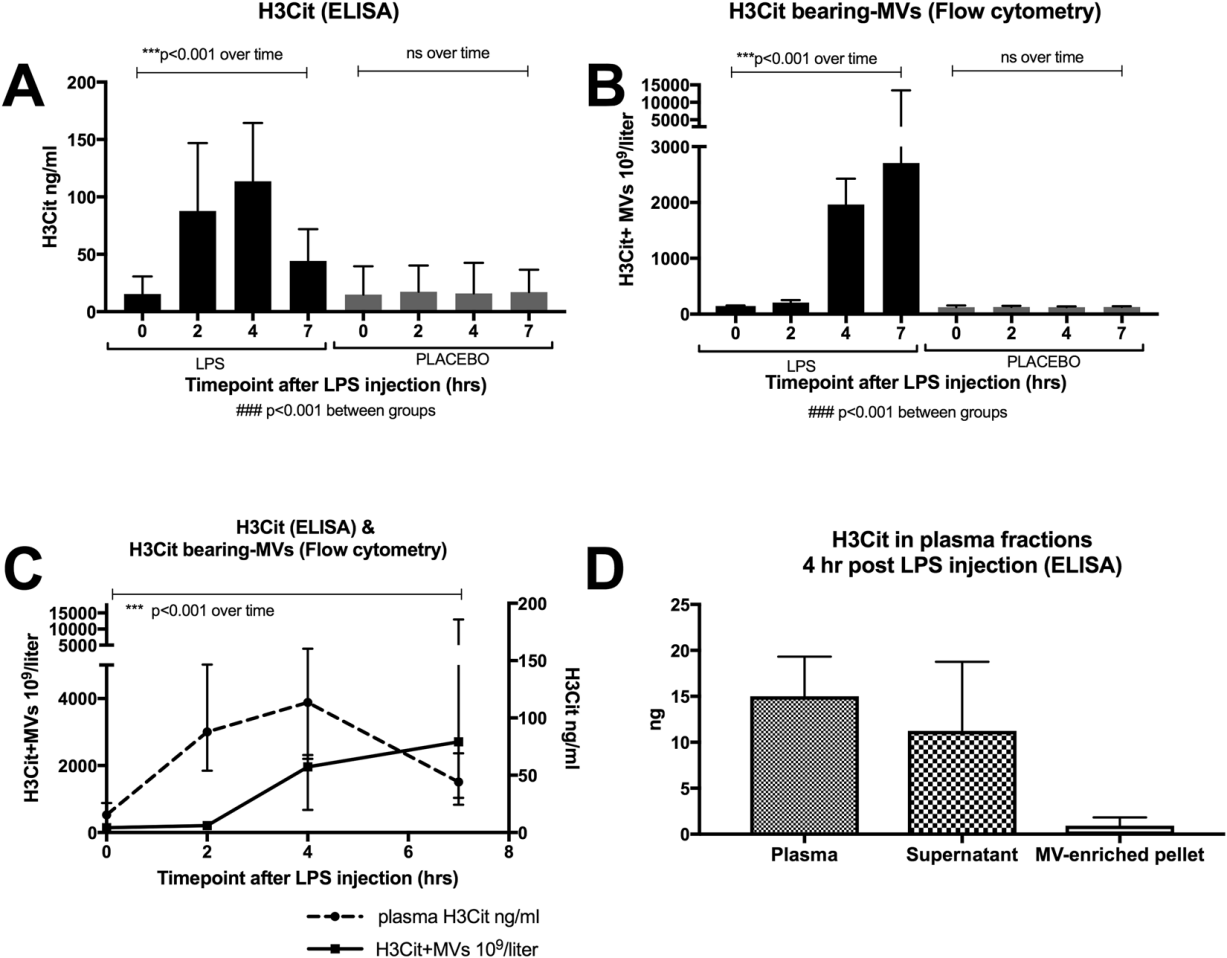
Circulating levels of H3Cit increased significantly after LPS injection in a human model of endotoxemia, comprising both an MV-bound and a non-MV-bound portion. (**A**) ELISA-detected levels of total plasma H3Cit increased 5-fold 2 hrs post LPS injection, peaked at 4 hrs, and approached baseline at 7 hrs. (**B**) Flow cytometry-detected levels of H3Cit-bearing MVs increased slightly but significantly at 2 hrs post LPS injection, and 13-fold at 4 hrs. The levels stayed elevated over the whole 7 hrs study period. (**C**) H3Cit-bearing MVs detected by flow cytometry (▬) displayed a delayed but sustained increase compared to ELISA-detection of total plasma H3Cit levels (---). (**D**) ELISA detection of H3Cit in MV-free supernatant and MV-enriched pellet 4 hrs post LPS injection comprised approximately 85% and 10% respectively of the total amount of H3Cit detected in plasma. Figure 1C and D display the LPS arm only. H3Cit; citrullinated histone H3, MVs; microvesicles, ELISA; enzyme-linked immunosorbent assay, LPS; lipopolysaccharide, time; change in levels of H3cit over the study time, between groups; LPS compared to placebo. Data are presented as median and interquartile range. N = 22 for all observations.

Using an ELISA assay, we determined plasma levels of H3Cit in a human model of endotoxemia (Fig. 1A). A five-fold increase in median plasma H3Cit levels was observed 2 hrs after LPS injection (p < 0.001), with a peak at 4 hrs. The levels decreased (p < 0.001) but remained elevated 7 hrs post LPS injection. No significant elevation was seen in the placebo arm (Fig. 1A). These results indicate an endotoxin-associated increase in circulating H3Cit, readily quantifiable with ELISA.

### H3Cit can be detected bound to MVs and account for a portion of the elevated levels of circulating H3Cit observed after LPS injection

To determine whether H3Cit can be detected bound to circulating MVs, we performed a flow cytometric assay quantifying H3Cit-bearing MVs in plasma (Fig. 1B). There was a slight but significant increase in circulating H3Cit-bearing MVs 2 hrs post LPS injection (p < 0.01), and the median level rose 13-fold by 4 hrs compared to baseline (p < 0.001). The increased levels sustained over the whole 7 hr study period (Fig. 1B). The MFI of H3Cit-bearing MVs demonstrated the same changes over time; i.e. slight but significant increase after 2 hrs post LPS injection (p < 0.05), followed by higher MFI levels at 4 and 7 hrs (p < 0.001) (Supplemental Fig S1). No significant differences in H3Cit-bearing MVs (both concentration and MFI) were seen in the placebo arm (Fig. 1B). The different dynamics found in the levels of circulating H3Cit, in the LPS arm, detected by ELISA and MV-bound H3Cit detected by flow cytometry are illustrated in Fig. 1C, suggesting a delayed but sustained elevation of MV-bound H3Cit. There was no difference in results depending on if LPS was administered day 1 or 2.

To further assess the portion of circulating H3Cit bound to MVs in our samples, we proceeded with ELISA-quantification of the levels of H3Cit in plasma 4 hrs post LPS injection after high-speed centrifugation, rendering an MV-free supernatant and an MV-enriched pellet. The amount of H3Cit detected in the MV-free supernatant comprised approximately 85% of the total amount of H3Cit detected in plasma, and the amount of H3Cit detected in the MV-enriched pellet, assumed to be bound to MVs, comprised approximately 10% of the total amount of H3Cit detected in plasma (Fig. 1D). Taken together, these results indicate that a minor but non-negligible portion of endotoxin-induced H3Cit-increase is bound to circulating MVs, and that this portion increases at a later stage, with a sustained elevation, compared to the total amount of H3Cit detected with ELISA. No difference was seen if LPS was administered day 1 or 2.

### H3Cit binds selectively to *in vitro* generated MVs expressing PS

**Figure 2:**
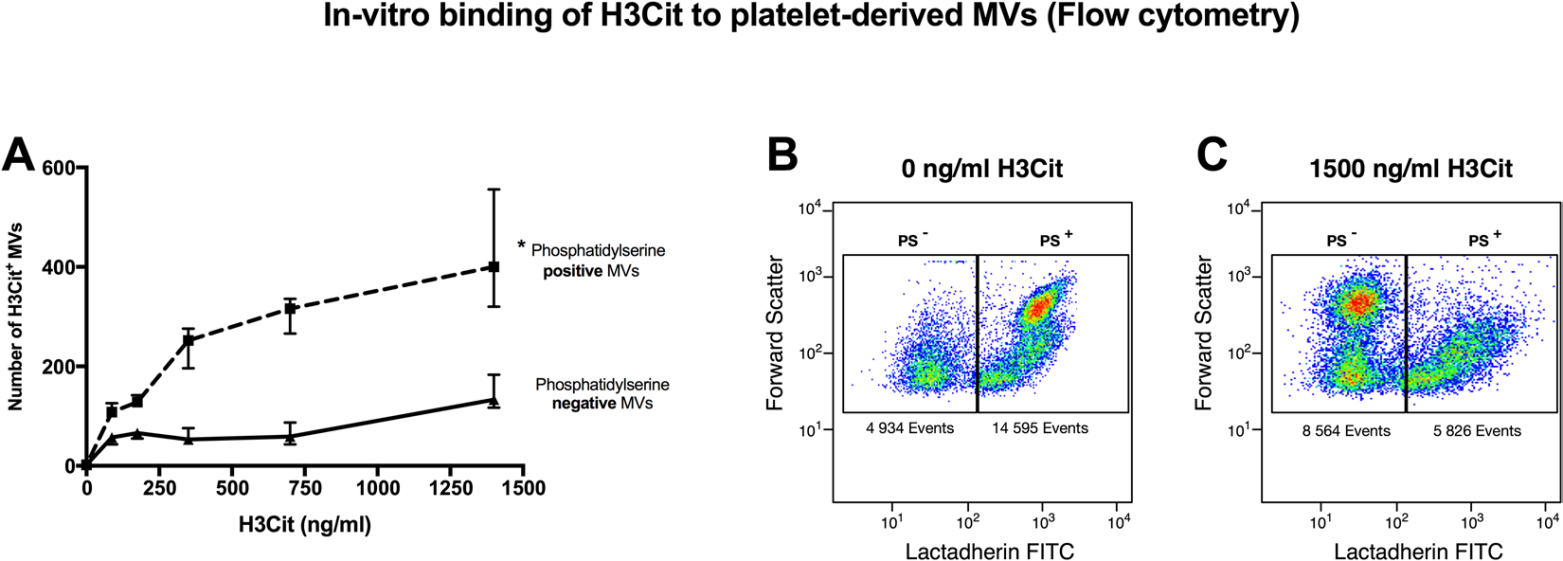
H3Cit binding to plaletet derived MVs (**A**) *In vitro* generated MVs were incubated with different concentrations of purified H3Cit (90 min, 37 °C, 0–1500 ng/ml). Co-expression of H3Cit and phosphatidylserine (PS) was investigated in the MV population. Results indicate a significant increase in H3Cit-bearing MVs only in the PS positive MV population (^*^p < 0.05) compared to PS negative MVs when incubated with an increasing concentration of H3Cit. (**B**) Representative flow cytometric plots of PS exposing MVs incubated with purified H3Cit; 0 ng/ml (**B**) and 1500 ng/ml (**C**). PS exposure was measured with FITC labeled lactadherin. Results demonstrate a higher number of MVs in the PS negative gate after H3Cit incubation. MVs; microvesicles, PS; phosphatidylserine, H3Cit; citrullinated histone H3, Data are displayed as median and range. (N = 3), *p < 0.05 represents the difference over time between PS positive MVs compared to PS negative MVs.

To study the susceptibility of H3Cit to bind to MVs and to assess whether this binding is in part a binding between H3Cit and PS exposed on MVs, we proceeded to incubate *in vitro* generated platelet derived MVs (PMVs) from healthy donor-blood with purified H3Cit (Fig. 2). By flow cytometry, MVs were first phenotyped based on PS exposure. The numbers of H3Cit-bearing MVs in each population (PS^+^ positive MVs and PS^−^ negative MVs respectively) were subsequently investigated at each concentration of H3Cit incubation (0–1500 ng/ml). Results demonstrated that H3Cit binds selectively to PS positive MVs in a concentration dependent manner as seen in Fig. 2. No increase could be detected in H3Cit-bearing MVs in the PS negative MV population. Taken together, this indicates a binding/complex formation between H3Cit and PS exposed on MVs, possibly through electrostatic forces between the positively charged H3Cit and negatively charged PS.

### Endotoxin-induced elevations of circulating H3Cit-bearing MVs are derived from both neutrophils and platelets, but H3Cit was detected predominately on neutrophil-derived MVs

**Figure 3:**
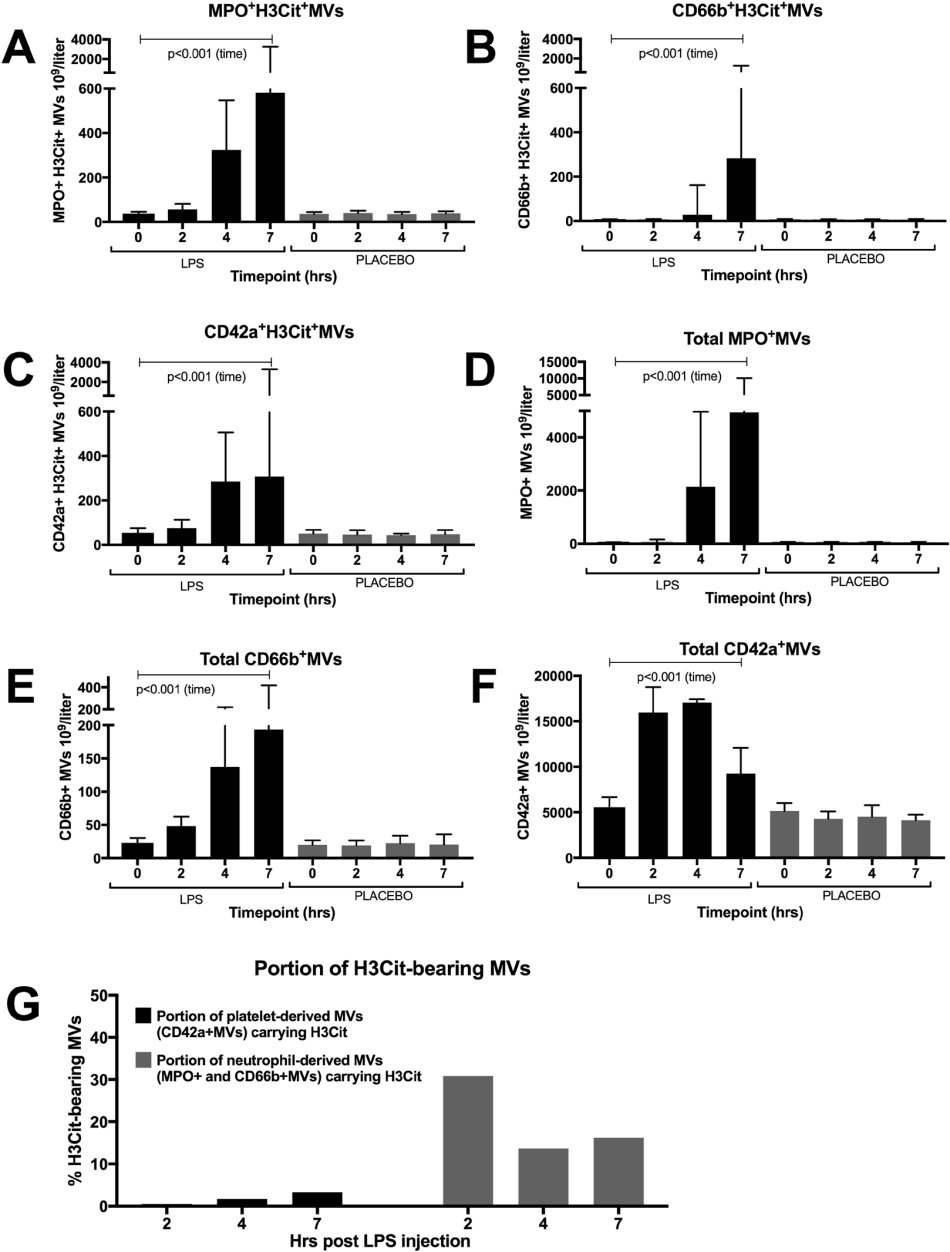
Phenotypic analyses of MVs indicated that H3Cit-bearing MVs were derived from both platelets and neutrophils. Although the majority of MVs were platelet derived, neutrophil derived MVs was the subpopulation with the largest portion of H3Cit-bearing MVs. H3Cit-bearing MVs exposed both neutrophil (MPO; **A** and CD66b; **B**) and platelet (CD42a; **C**) markers. Neutrophil derived MVs (MPO^+^MVs; **D** and CD66b^+^MVs; **E**) comprised a minority of the total amount of MVs detected in plasma compared to platelet derived MVs (CD42a^+^MVs; **F**). **G** Although the vast majority of MVs detected were platelet derived (CD42a^+^MVs), neutrophil derived MVs (MPO^+^MVs and CD66b^+^MVs) was the subpopulation with the largest portion of H3Cit bearing MVs. Figure 3G displays the LPS arm only. H3Cit; citrullinated histone H3, MVs; microvesicles, LPS; Lipopolysaccharide, time; change in levels of MVs over the study time in the LPS arm compared to placebo. Data are presented as median and interquartile range. No significant difference was seen if LPS was administered at 1^st^ or 2^nd^ study occasion. N = 22 for all observations.

Since H3Cit is released from neutrophils into the blood stream following neutrophil activation and NETosis, we investigated the presence of the neutrophil activation markers CD66b and MPO on the surface of the H3Cit-bearing MVs. Both CD66b- and MPO-positive H3Cit-bearing MVs increased significantly after LPS injection (p < 0.001), peaking at 7 hrs, with the largest increase in MPO positive H3Cit-bearing MVs. No significant increase was seen in the placebo arm. (Fig. 3A,B). As platelet-neutrophil interactions have been shown to drive NETosis in sepsis, we proceeded to investigate the surface presence of the platelet marker CD42a on the H3Cit-bearing MVs, revealing similar elevations of H3Cit-bearing MVs expressing CD42a after LPS injection (p < 0.001) (Fig. 3C).

In addition to H3Cit-bearing MVs, we investigated the total concentrations of MVs expressing MPO, CD66b and CD42a (regardless of H3Cit-exposure) in order to detect early dynamics in platelet and neutrophil activation following LPS injection. The vast majority of circulating MVs were platelet-derived over the whole 7 hr study period. In contrast to both H3Cit-bearing MVs and the subset of neutrophil-derived MVs, which peaked at 4–7 hrs, platelet derived MVs peaked as early as 2–4 hrs following LPS injection (Fig. 3D–F). This early and dramatic increase in platelet-derived MVs is in line with prior data^43^, indicating a rapid and substantial platelet response to LPS, most likely by TLR4 signalling pathway^44^. However, despite the substantially higher number of platelet-derived MVs, the majority of MVs carrying H3Cit were neutrophil-derived (Fig. 3A–C). In line with this, the portion of H3Cit-bearing MVs among neutrophil-derived MVs was substantially higher than the portion of H3Cit-bearing MVs among platelet-derived MVs (Fig. 3G). Although we cannot rule out an additional and concomitant source of H3Cit-bearing MVs, these results imply that at least a portion of the H3Cit-bearing MVs were derived from activated neutrophils, conceivably through NETosis. The small but significant portion of H3Cit-bearing platelet-derived MVs may support prior data on the role of platelets, platelet activation and platelet-neutrophil interaction in NET formation.

## Discussion

This is the first study to demonstrate dynamics and clear elevations of circulating H3Cit in a randomized and placebo-controlled human model of endotoxemia. These elevations were detected using two distinct assays; an ELISA and a flow cytometric assay. By flow cytometry, we furthermore demonstrate, to the best of our knowledge for the first time, that H3Cit can be detected bound to MVs. This not only allows for a rapid and robust assessment of *in vivo* NET formation in a large number of samples, but also proposes a possible mechanism by which NET components, such as H3Cit, may be disseminated throughout the vasculature.

Our human data on the increase in circulating H3Cit following LPS-injection is in line with prior studies presenting elevations of plasma H3Cit in murine models of LPS-induced inflammation^19,20,33^. Furthermore, a recent clinical study by Hampson *et al*.^34^ detected plasma H3Cit by western blot in septic patients. Interestingly, in the murine models presented by Pan *et al*.^33^, there were clear elevations of plasma H3Cit only in mice with LPS-induced shock but not in mice with haemorrhagic shock, and H3Cit was more responsive to endotoxemia than the clinically implemented septic biomarker procalcitonin and the inflammatory cytokines IL-1β and IL-6. These data imply that circulating H3Cit may be able to identify patients with sepsis. NETs are released rapidly following LPS stimulation^9^, supported by the detection of plasma H3Cit as early as 30 min post LPS-injection in the murine model by Pan *et al*. In our human model of LPS-induced endotoxemia, we were able to detect plasma H3Cit 2 hrs post LPS-injection. An even earlier rise can, however, not be ruled out, as the first samples were collected at 2 hrs.

The novel finding of H3Cit-bearing MVs was corroborated *in vitro* in a concentration dependent manner. This binding may likely, at least in part, be due to a complex formation between circulating H3Cit and PS exposed on MVs, possibly through electrostatic forces. The ELISA assay furthermore detected a small but non-negligible portion of H3Cit in MV-enriched pellet, corroborating an MV-bound population of H3Cit. Free histones are subject to rapid degradation in plasma, as shown by Ekaney *et al*.^45^, revealing a half-life of 4.6 min. However, H3Cit bound to DNA in nucleosomes/NET complexes may be protected against degradation. In like manner, complex formation between H3Cit and circulating MVs may well render the same protective effect and preservation in plasma. The binding of H3Cit to MVs may thus facilitate transportation throughout the vasculature and to distant organs. Although speculative, the relatively delayed and sustained elevations of circulating H3Cit-bearing MVs detected by flow cytometry compared to H3Cit detected by ELISA in our samples may possibly reflect a delayed binding between H3Cit and MVs following NET release. H3Cit carried by MVs may also have a prolonged lifetime due to a protective effect of MV-binding. Interestingly, Ekaney *et al*.^45^ also demonstrated that histone degradation reached a plateau after 30 min, with no further decline during the 6 hr observation. The authors speculate that the biological half-life of histones in plasma may be affected by the binding of histone fragments to other proteins protecting them from further degradation. MVs may thus serve as possible protecting carriers, although this is highly speculative and further studies investigating the effect of MV-binding on the biological activity of H3Cit are warranted.

The current study did not determine the source of H3Cit in plasma. However, considering the crucial role of histone citrullination in NETosis, and the large number of studies showing sepsis-induced elevations of various markers associated with NETs, such as cfDNA^11,15,22^, nucleosomes^23,24^, neutrophil elastase-DNA complexes^25^, MPO-DNA complexes^12^, and *in vitro* NET formation^9,13^, neutrophil activation and NETs seem a conceivable source. In support of this we show that the majority of H3Cit-bearing MVs were neutrophil-derived. As mentioned, platelets have been prescribed a central role in NET formation in sepsis. The phenotypic analysis of circulating MVs revealed that platelet derived MVs rose earlier than both neutrophil derived and H3Cit bearing MVs. In line with prior data this suggests that platelet MV formation is rapid following LPS stimulation^43^, but that neutrophil MV formation as well as the complex formation between H3Cit and MVs demands more time, perhaps reflecting a platelet-dependent NET formation. Of note, the MVs binding H3Cit in the *in vitro* experiments were platelet derived, and a portion of circulating H3Cit-bearing MVs expressed CD42a. Taken together, this supports the idea of platelet-derived MVs picking up H3Cit released from neutrophils in the circulation. However, although the presence of neutrophil- and platelet activation markers on the H3Cit-bearing MVs imply a neutrophil- and platelet activation and suggest that H3Cit may have bound to MVs during, or shortly after, NETosis, a complex binding unrelated to NETosis cannot be ruled out. Indeed, MPO is a highly positively charged secreted protein^29^, released by activated neutrophils and macrophages also in the absence of NETosis, and may subsequently bind to circulating MVs independent of a prior NET formation.

Regardless of the modality of dissemination throughout the vasculature, excess of circulating NET components, including histones, have been shown to have detrimental effects on the host. Several constituents of NETs have been implicated in the activation of coagulation^10^, and PAD4-mediated chromatin decondensation, rendering H3Cit, has been shown to be crucial for thrombus formation^14^. Extracellular histones have been demonstrated to contribute to macro- and microvascular thrombosis as well as epithelial and endothelial dysfunction, tissue injury and organ failure in murine models of sepsis^11,15–17^. In light of the harmful effects of NETs, promising NET inhibiting agents, such as DNase^13,15,21^ and PAD-inhibitors^18,20,33^ have been assessed in murine models, some of which have been shown to suppress circulating H3Cit levels with improved survival^18,20,33^. Apart from serving as a potential diagnostic and prognostic biomarker, H3Cit may thus also be useful in monitoring response to treatment, and may even become a target for new therapeutic strategies in the combat of sepsis and sepsis-associated complications.

There are certain limitations of the present study worth noting. In lack of commercially available assays to quantify plasma H3Cit, we used a tailor made sandwich ELISA, validated^36^ and implemented in recent studies on clinical samples^37,38,46^. We cannot, however, rule out possible uncertainties of the actual concentrations, such as an underestimation due to a possible competing step between histones and H3Cit in the capture step of the ELISA, or an incomplete citrullination of the *in vitro* citrullinated standard of H3Cit. The human model of LPS-induced endotoxemia is furthermore experimental, and the results must be regarded as such.

In conclusion, our results support earlier data on the pivotal role of NETs in sepsis by providing clear elevations of circulating H3Cit in a human model of endotoxemia. These elevations were detected by both an ELISA and a flow cytometry assay, providing two distinct methods to quantify a systemic NET burden. Furthermore, our results reveal that H3Cit can bind to MVs, proposing a mechanism by which H3Cit may be disseminated throughout the vasculature. Further research is now warranted to support these findings in a real-life clinical setting of sepsis. If confirmed, plasma H3Cit and H3Cit-bearing MVs may show to be promising blood biomarkers in the prediction and early diagnosis of sepsis as well as for prognosing its outcome.

## Materials and Methods

### Study design and study participants

**Figure 4:**
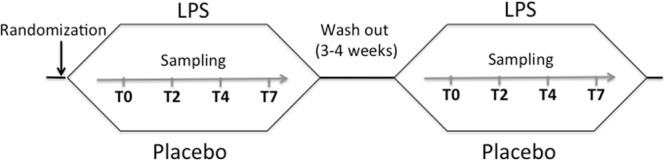
Study design. Study participants (N = 22) were randomized to intravenous injection of LPS or placebo (physiological saline) with a washout period of 3–4 weeks between interventions. Venous blood samples were obtained at baseline, 2, 4 and 7 hrs post injection (T0-T7). LPS: lipopolysaccharide 2 ng/kg body weight (*Escherichia coli* endotoxin).

The study was conducted at the Center for Clinical Research at Danderyd Hospital, Stockholm, Sweden and complied with the declaration of Helsinki. The study protocol was approved by the regional ethical review board in Stockholm, Sweden (registration number: 2014/1946-31/1). All study participants signed a written informed consent. The study protocol has been described previously^47^. Briefly, 22 healthy volunteers were included in the study (median age 23 (19–34) years; 9 women). A double-blinded, placebo-controlled, cross over design was used (Fig. 4). Study participants were randomly assigned to receive either an injection of LPS (*Escherichia coli* endotoxin, Lot H0K354 CAT number 1235503, United States Pharmacopeia, Rockville, MD, USA) at 2 ng per kg body weight or the same volume of placebo (physiological saline; 0.9% NaCl). The randomization was performed using coded envelopes. Study participants were assigned to the reverse intervention after a 3–4 week wash out period (Fig. 4).

### Sample collection

Blood samples were taken through an intravenous cannula at baseline, and then at 2, 4 and 7 hrs post injection of LPS/placebo. Citrated whole blood was centrifuged immediately at 2000 g for 20 minutes in room temperature (RT) to obtain platelet poor plasma (PPP). Samples were stored at −80 °C until further analysis.

#### Detection of plasma H3cit by ELISA

Plasma samples were thawed once on ice. H3Cit was quantified using a capture ELISA previously described and validated^36^. Briefly, H3Cit levels were obtained using an anti-histone antibody as capture antibody (Roche, Basel, Switzerland), and an anti-histone H3 citrulline antibody for detection (Abcam, Cambridge, UK). To assess the portion of MV-bound H3Cit in the samples, the same assay was performed on plasma after high-speed centrifugation (20 800 g, 45 min at RT), rendering an MV-free supernatant and an MV-enriched pellet.

#### Detection of plasma MVs by flow cytometry

Plasma samples were thawed once in water bath, 37 °C, and centrifuged at 2000 g for 20 minutes at RT. The supernatant was then re-centrifuged at 13000 g for 2 min at RT. Subsequently, 20 µl of the supernatant was incubated for 20 min in dark, with 5 µl of anti-histone H3 citrulline antibody Dylight 755 (Abcam, Cambridge, UK) for the detection of H3Cit-bearing MVs, CD42a FITC (Beckman coulter, Brea, CA, USA) for the detection of platelet-derived MVs, and CD66b APC (Beckman coulter, Brea, CA, USA) and myeloperoxidase PE (MPO) (Beckman coulter, Brea, CA, USA) for the detection of neutrophil-derived MVs. MVs were measured by flow cytometry on a Beckman Gallios instrument (Beckman coulter, Brea, CA, USA). The MV gate was determined by the use of Megamix-Plus FSC beads (0.3, 0.5 and 0.9 µm in size; BioCytex, Marseille, France). MVs were defined as particles less than 0.9 µm in diameter (forward scatter), see Supplemental Fig. S2. Conjugate isotype matched immunoglobulins without reactivity against human antigens were used as negative controls. Representative flow cytometry plots in Supplementary Fig. S3. In the present study, results are shown as numbers of MVs (MV counted x standard beads added/L)/standard beads counted (FlowCount, Beckman Coulter, CA, USA). In addition, mean fluorescence intensity (MFI) of H3Cit expression was also investigated (Supplemental Fig S1). The intra- and interassay coefficients of variation for MV measurement were less than 9% respectively.

#### Incubation of purified H3Cit with in vitro generated platelet-derived MVs

*In vitro* experiments were performed to investigate the binding properties of H3Cit to MVs. A high concentration of platelet-derived MVs was generated from fresh platelets obtained from healthy donor blood. Briefly, citrated whole blood was centrifuged at 200 g for 10 min at RT in order to obtain platelet-rich plasma (PRP). The PRP was then collected, and activated with platelet agonist ADP (35 µM, Roche, Basel, Switzerland) for 20 min at RT, rendering platelet-derived MVs. After activation, the PRP was centrifuged at 2000 g for 20 min at RT to separate platelets from the supernatant containing platelet-derived MVs. The supernatant was then re-centrifuged at 20800 g for 45 min at RT twice (phosphate-buffered saline wash in between). The supernatant was removed, and an MV-enriched pellet was obtained and aliquoted into three samples (n = 3). The MV-enriched samples were first labelled with anti-histone H3 citrulline and lactadherin in order to obtain baseline levels of H3Cit and PS exposure. Briefly, 20 µl of each sample was labelled with 5 µl of anti-histone H3 citrulline Dylight 755 and lactadherin FITC (Haematologic Technologies, VT, USA). After 20 min incubation in RT, the samples were measured by flow cytometry as described above. After baseline measurements, the MV-enriched samples were incubated with increasing concentrations of H3Cit peptide (abcam, Cambridge, UK), 0–1500 ng/ml, for 90 min at 37 °C. The samples were then again labelled with anti-histone H3 citrulline Dylight 755 and lactadherin FITC, and measured by flow cytometry. MVs were defined as particles less than 0.9 µm in size (forward scatter) and phenotyped based on their PS exposure and expression of H3Cit (Fig. 2).

#### Statistics

Mauchly´s test of sphericity was used to verify equal variance in the data. Normality of the residuals was tested by Shapiro-Wilks test. Skewed data were logarithmically transformed. Differences between the LPS and placebo arm over time were investigated with repeated-measures analyses of variance (ANOVA) or with a mixed model (Generalized Estimated Equation) for the cases where the data were skewed despite log-transformation (CD66b^+^H3Cit^+^MVs and H3Cit ELISA). The covariates time (change in levels over time) and trial day (day to receive LPS) were used in the model. A non-significant result for the covariat “trial day” was considered to demonstrate enough LPS wash out.

Due to the relatively small sample size, a Wilcoxon test (rather than a paired t-test) was used for all post hoc comparisons even when an ANOVA was used. A p-value < 0.05 was considered significant. Analyzes were performed using SPSS version 23 (IBM, Armonk, NY, USA). All data are presented as median and interquartile range (due to the non-normal distribution of some variables) to obtain a uniform presentation.

## Electronic supplementary material

Supplemental figures

## Data Availability

The datasets generated during and/or analysed during the current study are available from the corresponding author on reasonable request.
