# Supplementary material for: Circulating H3Cit is elevated in a human model of endotoxemia and can be detected bound to microvesicles
Source: Sci Rep. 2018 Aug 23;8:12641. doi: 10.1038/s41598-018-31013-4 (PMC6107669; doi:10.1038/s41598-018-31013-4)
Supplement: Supplementary file 1 — Supplemental figures [file 41598_2018_31013_MOESM1_ESM.docx]

**Supplementary information**

**Circulating H3Cit is elevated in a human model of endotoxemia and can be detected bound to microvesicles**

Sofie Paues Göranson, Charlotte Thålin, Annika Lundström, Lars Hållström, Julie Lasselin, Håkan Wallén, Anne Soop, Fariborz Mobarrez

**Supplemental figure S1**

**

**S. Fig 1. Circulating levels of H3Cit^+^ MVs increased significantly after LPS injection in a human model of endotoxemia**

Flow cytometry-detected levels of H3Cit-bearing MVs increased slightly but significantly at 2 hrs post LPS injection with increasing levels at 4 and 7 hours post LPS injection. H3Cit+ MVs measured as concentration (A) and as mean fluorescence intensity (MF) (B).
H3Cit; citrullinated histone H3, MVs; microvesicles, LPS; lipopolysaccharide, time; change in levels of H3cit over the study time. Data are presented as median and interquartile range N=22 for all observations.

**Supplemental figure S2**


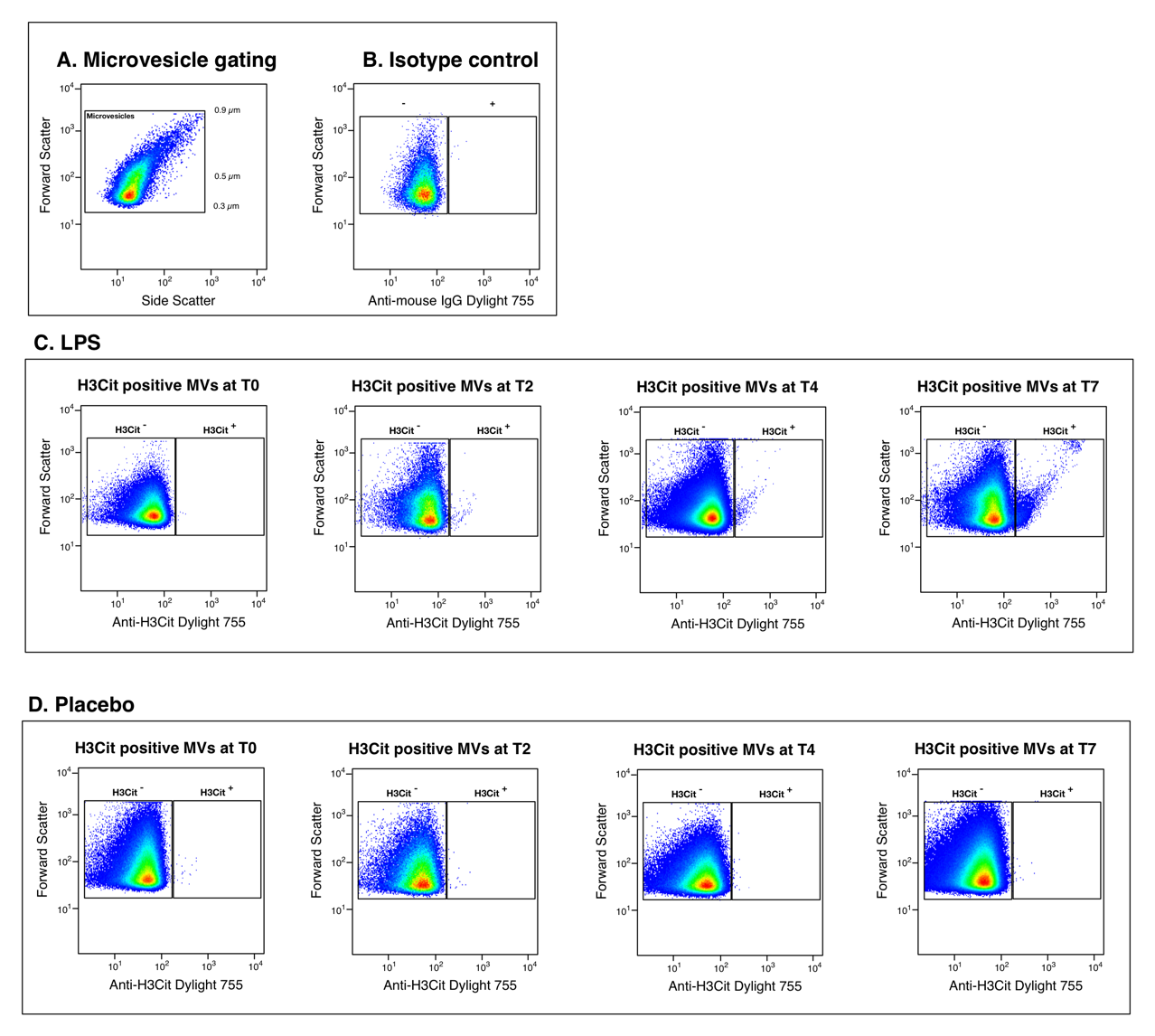


**S Fig 2**. **Schematic flow cytometric plots of** **A)** MV gating **B)** Isotype Control **C)** H3Cit positive MVs during LPS injection (0-7 hrs) and **D)** H3Cit positive MVs during placebo injection (0-7 hrs).

PS; phosphatidylserine, MVs; microvesicles; LPS; lipopolysaccharide; H3Cit; citrullinated histone H3.

**Supplemental figure S3**

**
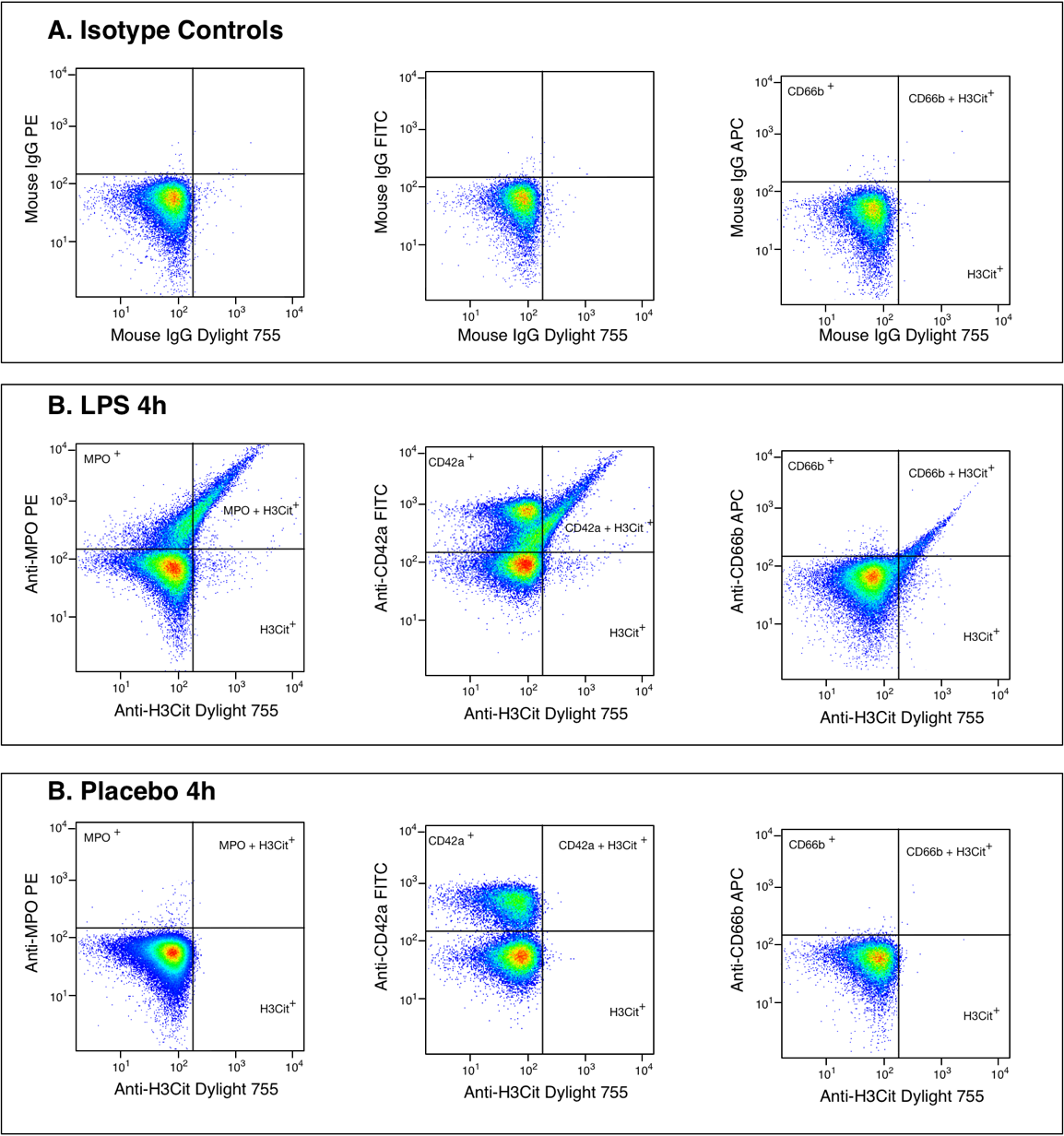
**

**S Fig 3**. **Schematic flow cytometric plots of H3Cit co-staining with neutrophil and platelet-derived MVs.**

**A)** Isotype Controls **B)** Co-staining of H3Cit with MPO, CD42a or CD66b during LPS injection (4h). **C)** Co-staining of H3Cit with MPO, CD42a or CD66b during placebo injection (4h)

LPS; lipopolysaccharide; H3Cit; citrullinated histone H3.
